# Mechanistic Disease Modeling to Inform Potential Strategies for Early Interventions with BACE inhibition

**DOI:** 10.1002/alz.094703

**Published:** 2025-01-09

**Authors:** Julie A Stone, Seth Robey, Matthew E. Kennedy, Stephen Duffull

**Affiliations:** ^1^ Merck & Co., Inc., Rahway, NJ USA; ^2^ MRL, Merck & Co., Inc., Boston, MA USA; ^3^ Certara, Princeton, NJ USA

## Abstract

**Background:**

The β‐secretase‐1 inhibitors (BACEi), including verubecestat, were extensively studied in prodromal to moderate AD and demonstrated early cognitive decline (negative effect) at doses achieving >50% inhibition of amyloid production. Questions remain as to whether BACEi may still have utility, if used earlier in disease and at lower levels of inhibition. A mechanistic model of the progression of Alzheimer’s disease was used to predict effects of alternative BACEi therapeutic approaches on disease progression.

**Method:**

The mechanistic model could holistically describe the available clinical data including observational trials and interventional trials with BACE inhibitors, anti‐amyloid mAbs, and anti‐tau antisense oligonucleotides. Varying inhibition levels (15‐ 56%) were simulated to predict amyloid PET, CSF Aβ42, CSF and plasma p‐tau, tau PET response. Delay‐times to BRAAK3‐4 region tau PET SUVr of 1.44 (MK‐6240; or 1.2 FTP) were calculated from simulations of intervention relative to untreated. Simulations of Down’s Syndrome populations (represented by increased production rate of amyloid) tested the impact on the dose and timing of the intervention needed to achieve benefits.

**Result:**

Sporadic AD simulations predicted that NFT progression could be delayed by decade(s) if started very early in disease (amyloid PET of 20‐50 centiloids) with moderate (33‐56%) levels of inhibition maintained for remainder of life. It’s unknown whether these inhibition levels have less cognitive decline than seen previously. Benefits were substantially reduced for later inventions (>60 centiloids), lower levels of inhibition or shorter durations of treatment (<10 years). Predicted biomarker timecourses suggested that separation from placebo within 4‐5 years would be demonstrable for CSF Aβ42 and amyloid PET, but p‐tau and tau PET alterations could take longer, especially for interventions starting at very low plaque loads. Limitations include the limited data from tau‐targeted approaches and on inhibition‐dependencies of the negative cognition effect, which create uncertainties in the predictions.

**Conclusion:**

Mechanistic model‐based simulations suggest that moderate level BACEi interventions very early in the process of amyloid plaque accumulations could lead to substantial delays in progression. The time to differentiation from untreated marker responses was long for tau‐based markers, suggesting that demonstration of effects during a typical trial timeline may be challenging.

